# Impact of Serum Nutritional Status on Physical Function in African American and Caucasian Stroke Survivors

**DOI:** 10.1155/2014/174308

**Published:** 2014-10-29

**Authors:** Monica C. Serra, Charlene E. Hafer-Macko, Frederick M. Ivey, Richard F. Macko, Alice S. Ryan

**Affiliations:** ^1^Department of Medicine, University of Maryland School of Medicine, 10 N Greene Street (BT/18/GR), Baltimore, MD 21201, USA; ^2^Geriatric Research Education and Clinical Center, Baltimore Department of Veterans Affairs Medical Center (VAMC), Baltimore, MD 21201, USA; ^3^Department of Neurology, University of Maryland School of Medicine, Baltimore, MD 21201, USA; ^4^Rehabilitation Research and Development's Maryland Exercise and Robotics Center of Excellence, Baltimore VAMC, Baltimore, MD 21201, USA

## Abstract

*Background*. The purpose of this study is to compare serum nutritional profiles in chronic stroke survivors to a representative sample of US Adults (NHANESIII) and determine whether these serum markers differed by race and impact physical function in stroke. *Methods*. Fasting serum samples were collected for analysis of lipids, uric acid, and albumin in 145 African American (AA) and 111 Caucasian (C) stroke survivors (age: 60 ± 1 years [mean ± SEM]). A six-minute walk was performed in a subset of stroke survivors (*N* = 134). *Results*. Triglycerides were higher and HDL-cholesterol and albumin lower in C than AA women stroke survivors (*P*s < 0.05). Uric acid was lower in C than AA stroke survivors (*P* < 0.05). Compared to NHANESIII, HDL-cholesterol, albumin, and hemoglobin generally were lower (*P*s < 0.05) and lipids were more favorable in stroke (*P*s < 0.01). Uric acid was related to six-minute walk performance among a subset of stroke survivors (*P* < 0.05). *Conclusion*. In stroke, racial differences exist with regard to serum nutritional risk, but these differences are similar to that observed in the general population. Regardless of race, nutritional risk appears elevated above that of the general population with regard to many of the serum markers. As a modifiable biomarker, uric acid should be monitored closely as it may provide insight into the functional risk of stroke survivors.

## 1. Introduction

Both suboptimal or excessive caloric intake and poor dietary quality affect nutritional risk and may hinder recovery from stroke. In as little as six months following discharge from an initial stroke incident, ~41% of survivors are at nutritional risk, based upon patient interviews regarding appetite, digestion, mobility, and swallowing difficulties [1]. Another study shows that 11% of stroke survivors with initial motor deficits and communication impairment still require feeding assistance six months after stroke [2]. Further, we have previously shown that well into the chronic phase of stroke recovery (>6 months), survivors are obese and have greater intramuscular fat relative to muscle area in their affected limb [3], indicating imbalanced dietary intake relative to energy expenditure. While these data suggest that poor caloric intake exists in chronic stroke, little data are currently available regarding diet quality in stroke.

Difficulties with speech and cognition may interfere with obtaining accurate dietary records to assess dietary quality in those chronically disabled by stroke. However, several serum biomarkers commonly found on general comprehensive chemistry panels are identified as useful indicators in the context of assessing nutritional status, providing a surrogate clinical indication of nutritional risk when dietary recall is not valid. For example, low iron is the main cause of low hemoglobin and many other nutrients (e.g., B_12_ and folic acid) can interfere with the formation of hemoglobin [4]. Additionally, the type of dietary fat consumed (e.g., saturated versus unsaturated fats [5]), as well as consumption of refined carbohydrates [6], may impact lipid and triglyceride concentrations in serum. Finally, low protein intake decreases albumin concentrations [7], while high intake of purine (a consequence of high animal protein consumption), saturated fat, vitamin C, alcohol, and fructose can all influence uric acid concentrations [8]. Further, serum concentrations of many of these markers have been linked to dietary intake through dietary and supplement recalls [9–11].

Many of these “nutritional” biomarkers are associated with measures of strength and maintenance of physical function in nonstroke populations. Specifically, anemia, secondary to low hemoglobin, is linked to reduced muscular strength and physical performance [12]. There are associative links demonstrated between low albumin and poorer grip strength [13], as well as high serum uric acid and higher hand and leg strength [14]. In stroke, physical limitations combined with nutritional inadequacies often manifest as a higher prevalence of obesity and intramuscular fat infiltration [3], thereby further impairing peak cardiorespiratory fitness and ambulatory function [15]. Hence, research determining whether nutritional biomarkers have utility in the context of better identifying nutritional imbalance after stroke or whether they might serve as clinical targets to gauge the benefits of lifestyle interventions in this population is of paramount importance.

We propose that nutritional serum biomarkers will be useful in determining nutritional and physical functional risk in persons with a history of stroke, especially as poststroke life expectancy continues to improve [16]. Although African Americans stroke survivors are more likely to report functional limitations than Caucasian stroke survivors [17], a recent study shows that approximately two-thirds of the racial disparity in stroke risk is explainable by African Americans' greater adherence to a dietary pattern high in fat, salt, and sugar [18]. The purpose of the current study was to compare serum “nutritional” profiles of stroke to recommended ranges and NHANES “controls,” as well as to determine the relationship of these serum profiles to body composition, strength, and physical function poststroke. We utilized serum samples from a large group of African American (AA) and Caucasian (C) chronic stroke survivors, with subsets tested for body composition, strength, and ambulatory function.

## 2. Materials and Methods

Two hundred fifty-six C and AA stroke survivors were recruited from the Baltimore area from March 2002 to January 2012 for participation in exercise rehabilitation studies. Volunteers for these intervention studies were at least six months removed from their ischemic stroke event (chronic phase), had residual hemiparetic gait deficits, and ranged in age from 40 to 85 years. Stroke latency was tracked for each of the 256 participants used in this baseline, cross-sectional analysis, enabling calculation of the mean time since stroke. All volunteers signed University of Maryland Institutional Review Board approved informed consent forms.

As part of screening for eligibility, volunteers underwent a health history and physical examination, which included height, weight, blood pressure, and a resting electrocardiogram. BMI categories were defined as underweight (<18.5 kg/m^2^), normal weight (18.5–24.9 kg/m^2^), overweight (25–29.9 kg/m^2^), and obese (≥30 kg/m^2^) [19]. Fasting serum samples also were collected for analysis of triglycerides, high density lipoprotein cholesterol (HDL-C), low density lipoprotein cholesterol (LDL-C), total cholesterol, hemoglobin, uric acid, and albumin. The results of serum analyses were compared to established recommendations for lipids [20], hematological indices [21], albumin [22], and uric acid [23] in middle-aged and older adults.

Dual-energy X-ray absorptiometry (DXA; *N* = 135) and computed tomography (CT; *N* = 78) scans were conducted in a subset of participants used in our cross-sectional comparison. Total body fat (%) and appendicular lean tissue mass (aLM: sum of lean mass in arms and legs) were assessed by DXA (DPX-L, DPX-IQ, and Prodigy; Lunar Radiation, Madison, WI). Skeletal muscle, fat, and low density lean tissue areas were quantified by mid-thigh CT scans (PQ 6000 Scanner; Picker International, Cleveland, OH and Somatom Sensation 64 Scanner; Siemens, Fairfield, CT) with data for the paretic leg reported.

VO_2_peak was measured to assess cardiorespiratory fitness using a graded treadmill test as previously described [24]. Mobility function was assessed during “self-selected” and “fastest-comfortable” pace 30-ft timed walks (3 trials at each speed), as well as based on six-minute walk distance. The walks were performed using the same assistive devices and/or orthoses that the volunteer used when walking at home. Repeated measures of concentric and eccentric hamstring muscle torque (measured in Nm) were obtained from the paretic leg using isokinetic dynamometry (Kin-Com 125AP, Chattex Group, Inc.) [25]. Hamstring torque generation (indicative of strength) was measured at 30°/sec over 60° of knee flexion/extension. The ratio of isokinetic torque to paretic leg lean tissue mass defined muscle quality (measured in Nm/kg).

Relevant to the current analysis, we selected all (*N* = 6, 782) C and AA adults (68% C and 47% male) aged 40–85 years from the NHANES III dataset who had validated hemoglobin, albumin, lipid, and uric acid measures, to serve as a reference population for the stroke participants included in our study. NHANES III is a nationally representative, cross-sectional study that examined the prevalence of major diseases and risk factors for these diseases in the United States. Full details of the study design can be accessed from the US Department of Health and Human Services [26].

Descriptive statistics were analyzed using SPSS (PASW Statistics, Version 18, Chicago, IL). Results were expressed as mean ± SEM. Student's *t*-tests were used to determine differences in volunteer characteristics and functional status. *χ* ^2^ tests were used to determine whether prevalence of categorical variables was different by race. Pearson correlations were used to assess relationships between nutritional markers and indicators of strength and function. Statistical significance was set at a two-tailed *P* < 0.05.

## 3. Results

### 3.1. Stroke Volunteer Characteristics and Functional Status

Mean latency since stroke was 3.9 ± 0.4 years. Sixty-four percent were on at least one lipid lowering medication. One percent were underweight, 30% were normal weight, 38% were overweight, and 31% were obese. None of these characteristics differed statistically by race. First, we analyzed whether racial differences in body composition and functional status were present within each sex (Table 1). BMI was greater in AA than C women (*P* < 0.01), but body fat percentage was not different. In contrast, BMI was not different between C and AA men, but body fat percentage was greater in C men (*P* < 0.01). Appendicular lean mass and mid-thigh muscle area were greater in both AA males and females than C men and women, respectively, (*P*s < 0.05), as anticipated. Mid-thigh fat and low density lean tissue area were similar by race within each sex, although there was a trend for greater mid-thigh fat area in AA versus C women (*P* = 0.06). There were no racial differences in peak fitness, with 96% of stroke survivors having “very poor,” 2% “poor,” and 1% “good” cardiorespiratory fitness by ACSM criteria [27]. Within each sex, no racial differences were observed with regard to strength, muscle quality, or physical function (six-minute walk distance and 30-foot walk times).

### 3.2. Nutritional Risk of Stroke Survivors by Race

**Figure 1:**
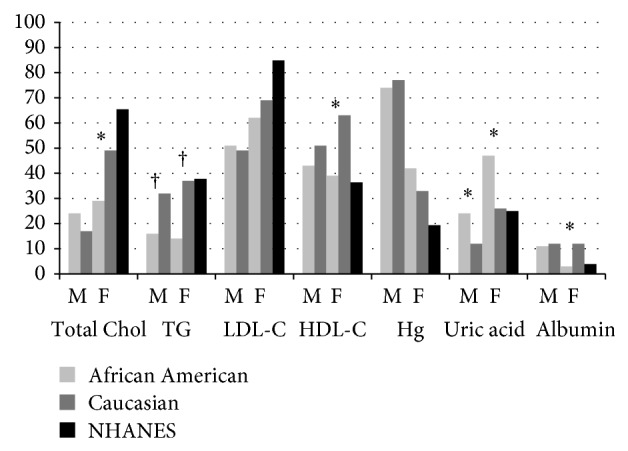
Bar graphs comparing the prevalence of serum concentrations outside of recommended ranges in male (M) and female (F) African American and Caucasian stroke survivors (^*^ *P* < 0.05, ^†^ *P* < 0.01 significantly different by race within a gender) compared to NHANES. Total Chol = total cholesterol, TG = triglycerides, and Hg = hemoglobin.

Mean nutritional profiles of stroke survivors by race are presented in Table 2 and racial differences in the prevalence of concentrations outside of recommended ranges may be viewed in Figure 1. Mean lipid profiles were similar between races in men; however, the prevalence of high triglycerides in C men was approximately twice that of AA men (*P* < 0.01). In women, triglyceride concentrations were 40% higher and high triglycerides were ~2.5 times more prevalent in C than AA (*P*s < 0.01). However, HDL-C levels were 11% lower in C women, with 24% more having low HDL-C than AA women (*P*s < 0.05). Although serum concentrations were not different, 20% more C versus AA women had high total cholesterol (*P* < 0.05). Uric acid concentrations were 10% lower in C versus AA stroke participants, regardless of sex (*P*s < 0.05). The prevalence of high uric acid in AA stroke participants was approximately two times that of C, across sexes (*P*s < 0.05). No racial differences were observed for hemoglobin concentrations. Serum albumin concentrations were 6% higher and the prevalence of low albumin was four times less in C versus AA women (*P*s < 0.05).

### 3.3. Comparison of Nutritional Risk between Stroke Survivors and Nonstroke Population (Table 2)

Compared to the nonstroke reference population, lipid concentrations were more favorable in both male and female AA and C individuals with stroke (*P*s < 0.01). However, male and female stroke participants of both races were more likely to have low HDL-C (*P*s < 0.05, except AA females where the mean was similar to NHANES) than NHANES. In men, uric acid concentrations were 5% lower in C stroke (*P* < 0.05) compared to C nonstroke from NHANES. Interestingly, uric acid concentrations were 9% greater among AA stroke women versus AA nonstroke NHANES women (*P* < 0.01), but there was no difference between C stroke and NHANES women. Across races and genders, hemoglobin and albumin concentrations were ~5–10% lower in stroke compared to NHANES (*P*s < 0.05).

### 3.4. Relationship of Serum Nutrition Concentrations to Body Composition and Function in Stroke

In determining whether nutritional markers were related to body composition, we observed that lipids were not related to aLM, muscle quality, mid-thigh muscle, intramuscular fat area, or total body fat across all stroke sex and race subgroups. Additionally, hemoglobin and albumin were not correlated with mid-thigh muscle area or aLM. Uric acid was related to BMI (*r* = 0.25; *N* = 256), aLM (*r* = 0.33; *N* = 130), mid-thigh muscle area (*r* = 0.32; *N* = 78), and mid-thigh low density lean tissue area (*r* = 0.34; *N* = 78) (*P*s < 0.01). When analyzed by race, these correlations were only present between uric acid and BMI (AA: *r* = 0.21, *P* < 0.01, *N* = 138; C: *r* = 0.25, *P* < 0.01, *N* = 105) and between uric acid and aLM (AA: *r* = 0.33, *P* < 0.01, *N* = 72; C: *r* = 0.24, *P* < 0.05, *N* = 63).

Next, we determined whether nutritional status related to strength and physical function. Hemoglobin, lipids, and albumin were not related to VO_2_peak, 30-foot walk times, or six-minute walk distance. However, there was a weak correlation between uric acid and six-minute walk time (*r* = 0.16, *P* < 0.05; *N* = 134), as well as a trend for a relationship between uric acid and concentric (*r* = 0.20, *P* = 0.07; *N* = 75) and eccentric (*r* = 0.20, *P* = 0.07; *N* = 75) hamstring contraction torque. These positive associations were not present when the data were analyzed by race or after controlling for aLM. Time since stroke was related to the six-minute walk distance (*r* = 0.23, *P* < 0.05; *N* = 87), but not to other nutritional, strength, or functional measures.

## 4. Discussion

This study is the first to characterize nutritional risk based on serum nutritional profiles in AA and C chronic stroke survivors. We used these measures, in part, to determine whether nutritional status based on biomarkers relates to body composition, strength, and physical function after stroke. Results suggest that racial differences exist with regard to serum nutritional profiles in stroke and that nutritional risk differs from that in the general US population. Further, we showed that uric acid is related to measures of body composition and function in chronic stroke survivors.

Several clinical trials have shown an association between high concentrations of serum cholesterol and stroke risk [28, 29], indicating the importance of these values as markers for nutritional risk. Further, increasing HDL-C is an important strategy for cardiovascular disease risk reduction [30]. When compared by race, triglycerides were higher in C versus AA men and women. Further, C women with stroke also had lower HDL-C. This is similar to that observed in the general population, with AA nonstroke adults presenting with more favorable lipid profiles than C nonstroke adults [31, 32]. Surprisingly, when compared to the NHANES population, both AA and C stroke survivors had more favorable lipid profiles, except for HDL-C, which was lower in those with stroke. The better lipid profile is likely the result of wider cholesterol lowering medication usage in stroke survivors. The prevalence of lipid lowering medication usage in the US among the general population increases with age from 19% in adults aged 40–64 to 39% in adults aged 65–74 [33], well below the 64% found among stroke participants assessed in the present study. Further, HMG CoA reductase inhibitor mediations have been shown to have therapeutic benefits that go beyond lipid lowering effects, including anti-inflammatory and antithrombotic properties [34]. Notably, drugs available for cholesterol management do not robustly raise HDL-C levels, perhaps explaining why chronic stroke participants had lower HDL-C, but better total cholesterol and triglycerides than the reference NHANES sample. Lipid subfraction concentrations for stroke in the current study are comparable to those previously reported in this population [35].

During the acute phase of stroke, ~50% of patients have hyperuricemia [36], but the overall prevalence declines to ~30% by the chronic phase of recovery according to our current measurements. Uric acid concentrations in the current study are consistent with those previously reported in chronic stroke survivors [37]. Further, the racial disparity of lower uric acid in C nonstroke adults also has been demonstrated previously [38]. Importantly, the higher mean uric acid levels observed in AA versus C female stroke survivors and AA reference women may represent a potentially important monitoring and intervention target in this stroke subgroup.

The role of uric acid as a protective or deleterious functional biomarker is controversial. Using a modified Rankin scale to assess function, it appears that stroke survivors with both low (<~4.7 mg/dL) and high (>~6.9 mg/dL) baseline uric acid levels have poorer functional outcomes assessed at a 12-month follow-up compared to those with levels between this range [39]. This implies an optimal uric acid range for promoting physical function in stroke survivors. We show a relationship of increasing uric acid and improved strength and physical function. This relationship was not present after controlling for aLM. Numerous studies have shown a relationship between uric acid and muscle mass [40, 41]. Thus, it appears that muscle mass is a mediating variable between uric acid level and physical function, justifying consideration when comparing uric acid with function. Using the 6.9 mg/dL cutoff for both men and women, only 20% of stroke survivors in the current study would qualify as having elevated uric acid. This may explain our failure to see a cut-point where uric acid became deleterious to physical function.

Similar to lipids and uric acid, albumin status also differed according to race in chronic stroke. The prevalence of low albumin was higher in AA versus C stroke participants. Additionally, compared to NHANES, mean concentrations of albumin, as well as hemoglobin, were lower in stroke. Similar mean hemoglobin and albumin profiles of the chronic stroke survivors in this study have previously been observed in acute stroke [42]. These markers have been positively linked to improved physical function and our data indicate the need for monitoring nutritional markers following stroke.

The results of this study should be interpreted in light of a few considerations. In particular, this study did not directly assess either caloric intake or quality of dietary intake, which may have provided insight as to how dietary patterns affect serum nutritional profiles and physical function in chronic stroke. However, this limitation is partially balanced by our unprecedented ability to phenotype serum nutritional profiles and functional status in a large sample of chronic stroke survivors. Additionally, the stroke participants from this study appear similar to previous reports in stroke with regard to nutritional profiles and functional status (e.g., average self-selected and fastest-comfortable 30-foot walking speeds) [43, 44], implying generalizability of our findings to the larger stroke population.

## 5. Conclusions

In general, serum nutritional values were outside of the recommended ranges in a large percentage of our stroke study population, indicating that nutritional risk remains ongoing, well into the chronic phase of stroke recovery. Racial differences appear to affect nutritional status in stroke survivors similar to that observed in the general population. However, regardless of race, nutritional risk appears elevated above that of the general population with regard to many of the serum markers, including HDL-C, hemoglobin, and albumin. Most of our selected serum nutritional markers did not predict our measures of functional capacity or strength after stroke. However, as a potentially modifiable biomarker through dietary modifications and with associations with physical function, uric acid should be closely monitored in the context of providing insight into nutritional and functional risk after stroke. Future studies should determine optimal methods for examining dietary intake patterns after stroke and how intake affects serum nutritional markers, as well as general health and function in this special population.

## Figures and Tables

**Table 1:** Demographic, strength, and functional data in chronic stroke survivors.

	African American males	Caucasian males	African American females	Caucasian females
Age (yrs)	61 ± 1 (79)	64 ± 1 (76)	61 ± 1 (66)	64 ± 2 (35)
BMI (kg/m^2^)	29 ± 1 (79)	28 ± 1 (76)	29 ± 1 (66)	26 ± 1^**^(35)
Total body fat (%)	28 ± 1 (37)	33 ± 1^**^(40)	42 ± 1 (35)	42 ± 1 (23)
aLM (kg)	27 ± 1 (37)	23 ± 1^**^(40)	18 ± 1 (35)	15 ± 1^**^(23)
Mid-thigh muscle area (cm^2^)	82 ± 4 (22)	65 ± 4^**^(19)	50 ± 4 (21)	44 ± 3^*^(16)
Mid-thigh fat area (cm^2^)	57 ± 5 (22)	67 ± 6 (19)	135 ± 14 (21)	100 ± 9 (16)
Mid-thigh LDLT area (cm^2^)	22 ± 1 (22)	24 ± 2 (19)	22 ± 2 (21)	18 ± 1 (16)
Six-min walk distance (m)	248 ± 20 (39)	213 ± 17 (38)	210 ± 19 (34)	174 ± 20 (23)
30-foot walk-self-selected pace (ft/sec)	1.9 ± 0.2 (42)	1.8 ± 0.1 (48)	1.7 ± 0.1 (45)	1.6 ± 0.2 (26)
30-foot walk-fastest pace (ft/sec)	2.7 ± 0.2 (42)	2.4 ± 0.2 (48)	2.5 ± 0.2 (45)	2.1 ± 0.2 (26)
Concentric torque at 30°/sec (Nm)	41 ± 6 (24)	32 ± 4 (24)	29 ± 5 (26)	21 ± 5 (17)
Eccentric torque at 30°/sec (Nm)	84 ± 9 (24)	74 ± 7 (24)	57 ± 9 (26)	42 ± 7 (17)
Concentric muscle quality (Nm/kg)	4.5 ± 0.7 (19)	4.2 ± 0.5 (18)	4.0 ± 0.8 (22)	4.0 ± 0.9 (16)
Eccentric muscle quality (Nm/kg)	9.5 ± 1.0 (19)	9.5 ± 0.9 (18)	8.2 ± 1.2 (22)	7.7 ± 1.4 (16)
VO_2_peak (mL/kg/min)	16.3 ± 0.8 (47)	15.6 ± 0.7 (46)	11.6 ± 0.8 (42)	12.1 ± 0.7 (24)

Mean ± SEM (*N*). aLM: appendicular lean mass; LDLT: low density lean tissue. ^*^ *P* < 0.05; ^**^ *P* < 0.01: significantly different from African American stroke survivors of the same sex.

**Table 2:** Serum nutritional profiles of African American NHANES versus stroke survivors.

	Reference value	African American		Caucasian	
		Stroke survivors	NHANES	Stroke survivors	NHANES
Total cholesterol (mg/dL)					
Males	<200	170 ± 5 (*N* = 79)	211 ± 1^‡^	171 ± 6 (*N* = 76)	212 ± 1^‡^
Females	<200	188 ± 5 (*N* = 66)	221 ± 1^‡^	195 ± 6 (*N* = 35)	228 ± 1^‡^
Triglycerides (mg/dL)					
Males	<150	107 ± 7 (*N* = 79)	142 ± 4^‡^	121 ± 7 (*N* = 76)	171 ± 3^‡^
Females	<150	98 ± 6 (*N* = 66)	126 ± 3^‡^	137 ± 10^**^ (*N* = 35)	163 ± 3
LDL-C (mg/dL)					
Males	<100	105 ± 4 (*N* = 79)	136 ± 2^‡^	106 ± 6 (*N* = 76)	137 ± 1^‡^
Females	<100	114 ± 4 (*N* = 66)	139 ± 2^‡^	119 ± 6 (*N* = 35)	140 ± 1^‡^
HDL-C (mg/dL)					
Males	>40	43 ± 2 (*N* = 79)	52 ± 1^‡^	40 ± 1 (*N* = 76)	45 ± 1^‡^
Females	>50	54 ± 2 (*N* = 66)	57 ± 1	48 ± 2^*^ (*N* = 35)	55 ± 1^‡^
Hemoglobin (g/dL)					
Males	>14	13.1 ± 0.2 (*N* = 39)	14.1 ± 0.04^‡^	13.4 ± 0.3 (*N* = 26)	14.8 ± 0.03^‡^
Females	>12	12.2 ± 0.31 (*N* = 20)	12.6 ± 0.04	12.7 ± 0.46 (*N* = 10)	13.5 ± 0.02^†^
Uric acid (mg/dL)					
Males	>7	6.36 ± 0.17 (*N* = 79)	6.40 ± 0.05	5.75 ± 0.17^**^ (*N* = 73)	6.06 ± 0.03^†^
Females	>6	5.78 ± 0.20 (*N* = 66)	5.29 ± 0.05^‡^	5.20 ± 0.26^*^ (*N* = 35)	5.04 ± 0.03
Albumin (g/dL)					
Males	>3.5	3.9 ± 0.03 (*N* = 79)	4.0 ± 0.01^‡^	3.9 ± 0.05 (*N* = 79)	4.2 ± 0.01^‡^
Females	>3.5	3.7 ± 0.04 (*N* = 66)	3.9 ± 0.01^‡^	3.9 ± 0.03^**^ (*N* = 66)	4.0 ± 0.01^†^

Mean ± SEM. ^*^ *P* < 0.05; ^**^ *P* < 0.01: significantly different from African American stroke survivors. ^†^ *P* < 0.05; ^‡^ *P* < 0.01: significantly different from stroke survivors of the same race.
